# Hypertensive Emergency With End-Organ Damage and Multifocal Intracranial Lesions Mimicking Disseminated Metastatic Disease: A Case Report

**DOI:** 10.7759/cureus.107515

**Published:** 2026-04-21

**Authors:** Betul Ozdemir Ozturk

**Affiliations:** 1 Internal Medicine, Barking, Havering and Redbridge University Hospitals NHS Trust, London, GBR

**Keywords:** acute kidney failure, end-organ damage, end-stage renal disease (esrd), hypertensive, hypertensive crises, hypertensive emergencies, iga nephropathy, neuro-imaging, renal failure, vasogenic brain edema

## Abstract

Hypertensive emergency is a life-threatening condition characterized by severe elevation in blood pressure with evidence of acute end-organ damage. Neurological involvement may present with imaging findings that mimic intracranial malignancy, posing a significant diagnostic challenge.

A 41-year-old man with presumed IgA nephropathy and poor adherence to antihypertensive therapy presented with a 10-day history of headache, vomiting, and progressive visual disturbance. On admission, he was found to have severe hypertension (systolic blood pressure >200 mmHg) with evidence of end-organ damage, including bilateral retinal hemorrhages, exudates, macular edema, and acute-on-chronic renal failure with markedly elevated creatinine levels. Initial CT of the head demonstrated a right cerebellar hypodensity. Subsequent MRI of the head revealed multifocal intracranial lesions involving the cerebellum, temporal cortex, caudate nucleus, and medulla, raising concern for disseminated metastatic disease. However, contrast-enhanced CT of the chest, abdomen, and pelvis did not identify a primary malignancy. Follow-up contrast-enhanced MRI head performed on day 12 demonstrated marked interval resolution of the lesions, with only minimal residual abnormalities, findings not in keeping with metastatic disease and interpreted as a resolving inflammatory or infectious process.

Hypertensive emergency may be associated with reversible intracranial abnormalities that closely mimic metastatic disease. Careful clinicoradiological correlation, along with lesion improvement following blood pressure control, may support diagnostic clarification and help avoid unnecessary investigations.

## Introduction

Hypertensive emergency is defined as a critical elevation in arterial blood pressure (generally ≥180/120 mmHg) accompanied by acute hypertension-mediated injury to target organs [1]. Such presentations commonly involve the heart, kidneys, or brain and require prompt recognition and management. In the brain, cerebral autoregulation maintains stable cerebral blood flow across a range of blood pressures; however, when these limits are exceeded, increased hydrostatic pressure may disrupt the blood-brain barrier, leading to fluid extravasation and vasogenic edema [2]. This mechanism may contribute to the neuroimaging findings observed in hypertensive emergencies, which may be reversible with appropriate management.

Posterior reversible encephalopathy syndrome (PRES) is a clinicoradiological condition associated with severe hypertension and renal dysfunction, characterized by reversible vasogenic edema on neuroimaging [2,3]. Clinical presentation may vary widely, ranging from mild symptoms to life-threatening neurological complications [3]. However, not all hypertension-related brain injury conforms to classical PRES patterns, and imaging findings may be non-specific or misleading. Such imaging findings may mimic intracranial malignancy, potentially leading to diagnostic uncertainty.

A 41-year-old man with advanced renal failure presented with a hypertensive emergency and multifocal intracranial lesions initially suggestive of disseminated metastatic disease. Subsequent interval imaging demonstrated marked resolution of these abnormalities, highlighting a significant diagnostic challenge.

## Case presentation

A 41-year-old man with a background of presumed IgA nephropathy and progressive chronic kidney disease presented with a 10-day history of persistent occipital headache, nausea, vomiting, and progressive visual disturbance. He reported recent non-adherence to prescribed antihypertensive therapy for approximately one to three months and had intermittently used non-steroidal anti-inflammatory drugs for symptomatic relief.

He was initially evaluated by an optician, who identified bilateral retinal hemorrhages, exudates, and macular edema. He was subsequently referred to the hospital due to severe hypertension. On arrival, his systolic blood pressure exceeded 200 mmHg, with recorded readings of 207/139 mmHg and 208/150 mmHg. He denied limb weakness, sensory deficits, seizures, chest pain, or balance disturbance. Neurological examination was unremarkable, demonstrating normal gait, full limb power, absence of dysmetria or cerebellar signs, intact cranial nerves, and no focal neurological deficit.

**Figure 1:**
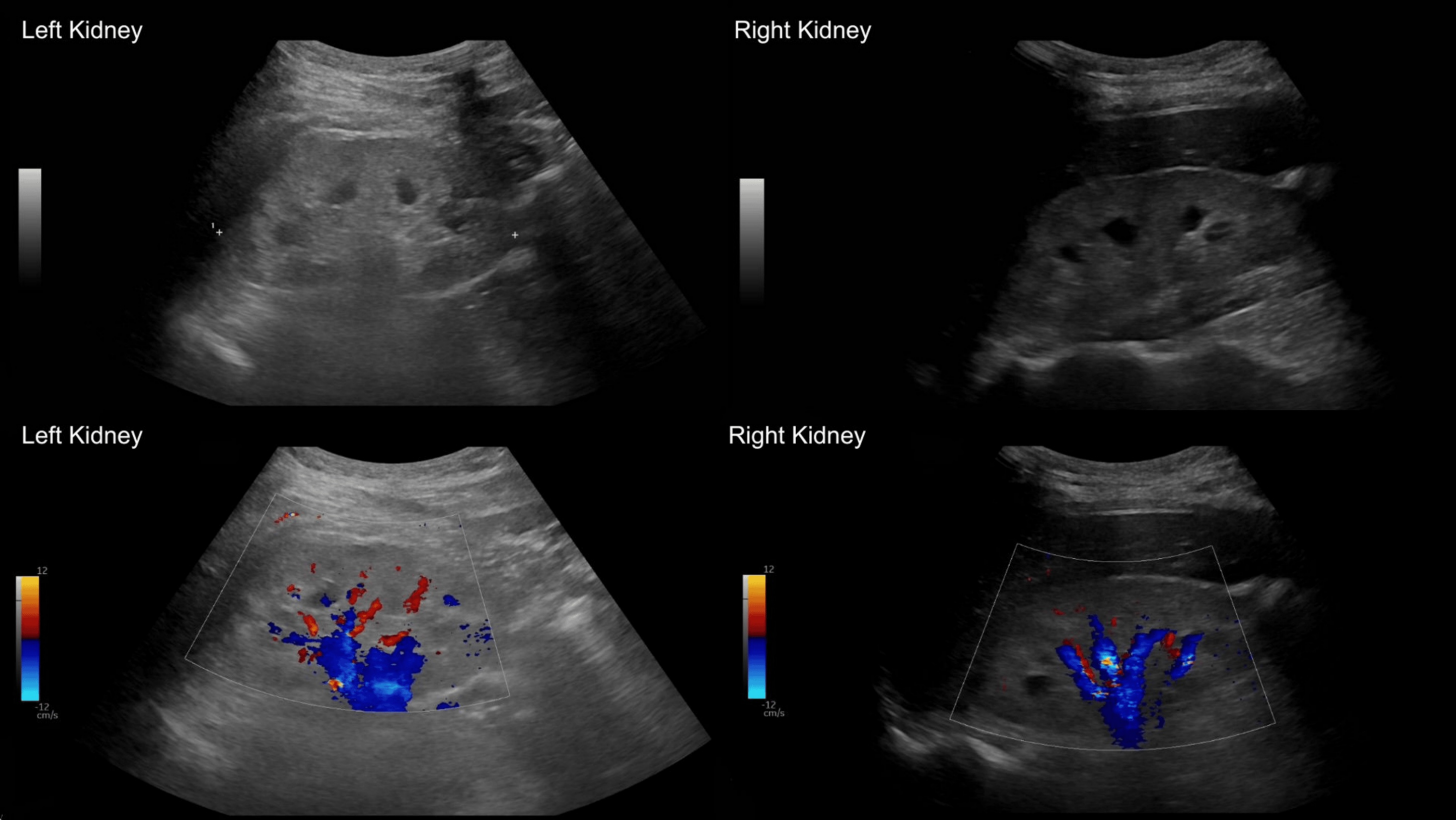
Renal ultrasound Ultrasound images of both kidneys demonstrate increased cortical echogenicity.

**Figure 2:**
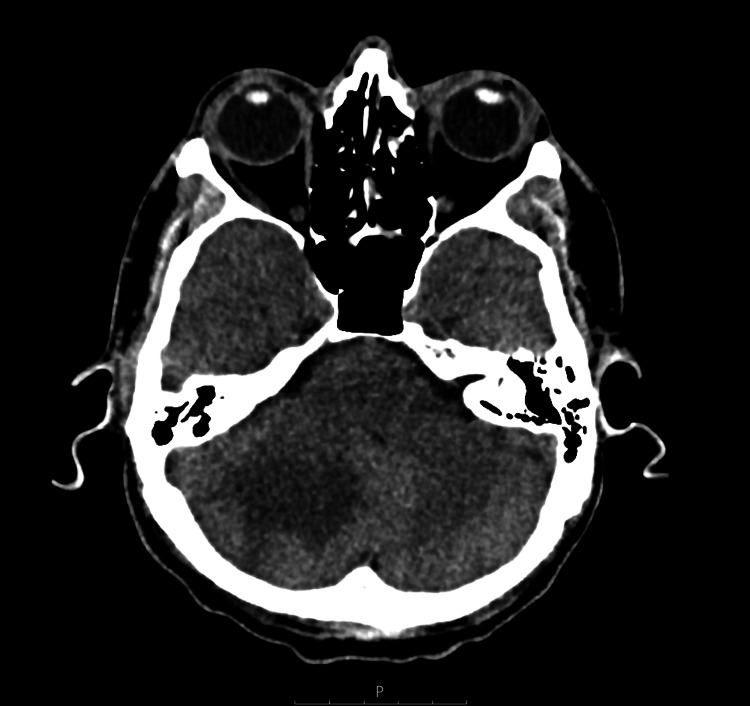
Non-contrast CT head reconstruction showing a focal area of low attenuation within the right cerebellar hemisphere

His renal history was notable for a progressive decline in kidney function over several years, with creatinine rising from 102 µmol/L in 2023 to 151 µmol/L in 2024, 302 µmol/L in 2025, and 743 µmol/L at presentation in 2026. Laboratory findings are summarized in Table 1. Renal ultrasound demonstrated bilaterally increased cortical echogenicity, consistent with renal parenchymal disease, without evidence of obstruction (Figure 1). Given the presence of severe hypertension and evidence of end-organ damage, a non-contrast CT scan of the head was performed, demonstrating an area of low attenuation within the right cerebellar hemisphere, raising concern for either acute infarction or an underlying lesion (Figure 2).

**Table 1:** Comparison of selected laboratory parameters at admission and during hospitalization

Parameter	Admission	Hospital Day 13	Reference Range
Sodium	137	138	133-146 mmol/L
Potassium	4.8	4.6	3.5-5.3 mmol/L
Urea	26.6	14.7	2.5-7.8 mmol/L
Creatinine	743	465	59-104 µmol/L
Estimated glomerular filtration rate (eGFR)/1.73m^2^	7	13	>60 mL/min/1.73 m²
Total bilirubin	5	<3	1-21 µmol/L
Alanine aminotransferase (ALT)	12	10	<41 U/L
Alkaline phosphatase (ALP)	88	77	30-130 U/L
C-reactive protein	1	3	<5 mg/L

**Figure 3:**
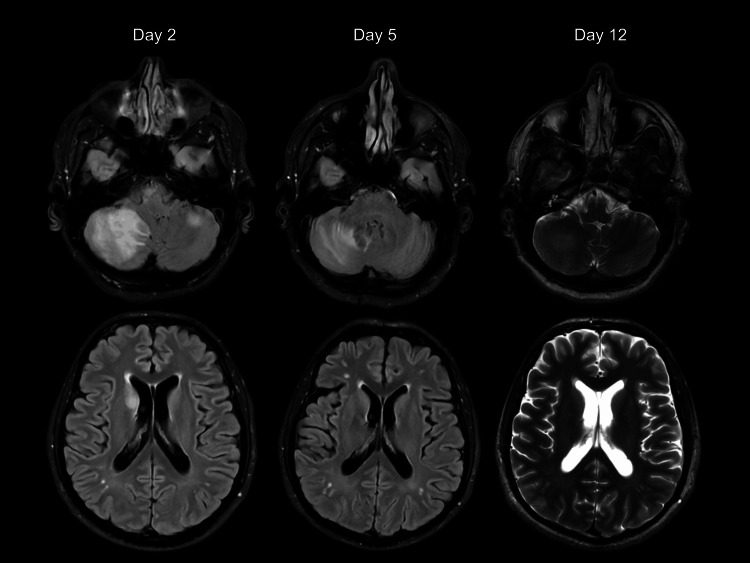
Serial MRI head demonstrating interval resolution of multifocal intracranial lesions Serial axial MRI head (T2-weighted and FLAIR sequences on days 2 and 5 and contrast-enhanced T2-weighted sequences on day 12) demonstrates progressive interval resolution of multifocal intracranial lesions. Initial non-contrast imaging shows a right cerebellar lesion with vasogenic edema and additional multifocal involvement. Follow-up imaging demonstrates gradual resolution, with improvement by day 12. FLAIR: fluid attenuated inversion recovery

MRI of the head performed on day 2 demonstrated a focal lesion in the right cerebellar hemisphere with associated moderate-to-severe vasogenic edema. Additional mild edema was noted in the left cerebellum, left posterior temporal cortex, and right caudate nucleus, with possible involvement of the ventral medulla (Figure 3). There was no diffusion restriction or hemorrhage. These findings were considered suspicious for a disseminated neoplastic process. During admission, blood pressure was gradually controlled. A repeat MRI of the head performed on day 5 demonstrated interval improvement, with reduced edema in the right cerebellum and resolution of previously noted lesions in the right caudate nucleus and left parieto-occipital region, although the overall appearance remained suspicious for metastatic disease (Figure 3). In view of these findings, further imaging with contrast-enhanced CT of the chest, abdomen, and pelvis was performed, which did not demonstrate any primary malignancy. A contrast-enhanced MRI head performed on day 12 demonstrated marked interval resolution of the lesions, with only a small residual focus in the right cerebellum showing minimal enhancement and mild residual edema (Figure 3). Previously identified lesions had resolved. These findings were not in keeping with metastatic disease and were interpreted as a resolving inflammatory or infectious process. The patient was initially managed in the intensive care setting and received continuous renal replacement therapy in the context of renal failure. Blood pressure was managed with intravenous and oral antihypertensive therapy, achieving gradual control. Following stabilization, he was referred for further nephrology input and long-term renal replacement planning.

## Discussion

Multisystem manifestations of severe hypertension may create a diagnostically challenging clinical picture, particularly when radiological findings mimic intracranial malignancy. In this case, retinal, renal, and radiological central nervous system findings contributed to initial diagnostic uncertainty. The presence of severe blood pressure elevation in conjunction with hypertensive retinopathy and advanced renal dysfunction confirms hypertension-mediated end-organ damage rather than isolated severe hypertension. The retinal findings observed in this case, hemorrhages, exudates, and macular edema, are in keeping with features of hypertensive retinopathy in the context of uncontrolled hypertension [4].

PRES is typically characterized by parieto-occipital edema [5]. However, a broader spectrum of involvement has been described, with lesions extending beyond the parieto-occipital regions to include the frontal and temporal lobes, as well as atypical areas such as the cerebellum, basal ganglia, deep white matter, brainstem, and thalamus [5-7].

In this case, the multifocal distribution of lesions involving the cerebellum, temporal cortex, caudate nucleus, and possible medullary structures created a radiological pattern highly suggestive of metastatic disease. Such a differential diagnosis is appropriate when imaging is assessed in isolation. This diagnostic dilemma has been described in the literature, where PRES may mimic malignancy [8,9]. However, the absence of an identifiable primary malignancy on cross-sectional imaging, combined with the rapid and near-complete resolution of lesions on follow-up MRI without oncological treatment, strongly argues against a neoplastic process. Metastatic lesions would not be expected to regress in this manner in the absence of targeted therapy.

The combination of severe uncontrolled hypertension, advanced renal impairment, and recent non-adherence to antihypertensive therapy provides a biologically plausible setting for failure of cerebral autoregulation with endothelial dysfunction [3]. In this context, abrupt elevations in blood pressure may exceed the autoregulatory capacity of cerebral vessels, resulting in dysregulation of cerebral blood flow, blood-brain barrier disruption, and vasogenic edema [3,7].

Chronic kidney disease is associated with an increased risk of PRES, with risk increasing as renal function declines, although the independent contribution of renal dysfunction remains uncertain [10]. Although PRES represents a well-recognized entity associated with these mechanisms, the findings in this case do not allow a definitive diagnosis of PRES. The radiological interpretation favored a resolving inflammatory or infectious process. This highlights a notable diagnostic difficulty, as imaging findings in a hypertensive emergency may overlap with a range of alternative pathologies.

A key feature in this case was the marked interval resolution demonstrated on serial imaging, which is not in keeping with a progressive or infiltrative process. This highlights the limitations of relying on a single imaging time point when interpreting multifocal radiological findings, particularly in the absence of corresponding neurological deficits, and emphasizes the importance of interval imaging in clarifying the nature of intracranial lesions.

From a clinical perspective, this case emphasizes the importance of careful clinicoradiological correlation in patients presenting with severe hypertension and atypical multifocal brain lesions, particularly in the context of renal dysfunction. Importantly, severe hypertension may produce neuroimaging findings that closely mimic intracranial malignancy, representing a potential diagnostic pitfall. Awareness of this potential overlap may help avoid misinterpretation as metastatic disease, prevent unnecessary investigations, and facilitate timely management of hypertensive emergencies to reduce the risk of further end-organ damage. Furthermore, this case highlights the potential for lesion reversibility following effective blood pressure control, which may support diagnostic clarification in appropriate clinical contexts.

## Conclusions

Hypertensive emergency may present with reversible multifocal intracranial abnormalities that can mimic disseminated metastatic disease, particularly in patients with predisposing factors such as chronic kidney disease. Although the imaging findings were initially highly suggestive of malignancy, their rapid interval resolution on follow-up imaging was not in keeping with a neoplastic process and was interpreted as a resolving inflammatory or infectious change. This case highlights an important diagnostic pitfall and suggests that improvement following blood pressure control may be helpful in supporting diagnostic clarification. Careful clinicoradiological correlation and follow-up imaging may play an important role in avoiding misdiagnosis and guiding appropriate management.
